# The development of effective behaviour change interventions to support the use of malaria rapid diagnostic tests by Tanzanian clinicians

**DOI:** 10.1186/1748-5908-9-83

**Published:** 2014-06-26

**Authors:** Clare IR Chandler, Judith Meta, Célia Ponzo, Fortunata Nasuwa, John Kessy, Hilda Mbakilwa, Ane Haaland, Hugh Reyburn

**Affiliations:** 1 Department of Global Health and Development, London School of Hygiene & Tropical Medicine, 15-17 Tavistock Place, London WC1H 9SH, UK; 2 Joint Malaria Programme, Kilimanjaro Christian Medical Centre, PO Box 2228, Moshi, Tanzania; 3 Nutrition and Reproductive Health Unit, Ministry of Health, PO Box 52, Mont Fleuri, Republic of Seychelles; 4 Institute of Health and Society, University of Oslo, PO Box 1130, Blindern NO-0318 Oslo, Norway; 5 Department of Disease Control and Vector Biology, London School of Hygiene & Tropical Medicine, Keppel Street, London WC1E 9HT, UK

**Keywords:** Complex intervention, Malaria, Rapid diagnostic tests, Prescribing behaviour, Clinical practice, Behaviour change theory, Evidence-based research, Formative research

## Abstract

**Background:**

Parasitological confirmation is now recommended for all cases of suspected malaria. The roll-out of rapid diagnostic tests (RDTs) is hoped to enable this goal in low resource settings through point of care testing. However, simply making RDTs available has not led to high uptake of the tests or adherence to results by clinicians, with malaria continuing to be overdiagnosed in many settings. We undertook to design an evidence-based intervention package that would be sufficient to support the introduction of RDTs at dispensaries in Tanzania, to be evaluated through the Targeting Artemisinin Combination Therapy (TACT) cluster randomised controlled trial.

**Methods:**

We describe five steps in our intervention design: formative research, review of existing evidence and theory, a workshop to define the intervention approach and content and results of formative research, engagement with behaviour change theory and literature, detailed design of intervention materials and piloting and pretesting of intervention materials. This involved fieldwork with a total of 19 health workers and 212 community members in northeast Tanzania.

**Results:**

The formative research suggested that RDTs were a potential source of conflict in the health worker-patient interaction, but that health workers used various techniques to resolve this, including provision of antimalarial drugs for RDT-negative patients. Our reviews showed that evidence was mixed regarding the effectiveness of different methods and theories to support change in prescribing practice. Our design process is presented, drawing from this collective evidence. We describe the final TACT intervention package (including interactive small group workshops, feedback text messages, motivational text messages and patient information leaflets and posters) in terms of its programme theory and implementation theory.

**Conclusions:**

Our study suggests that evidence-based design of complex interventions is possible. The use of formative research to understand malaria overdiagnosis in context was central to the design of the intervention as well as empirical research to test materials and methods prior to implementation. The TACT interventions may be appropriate for other settings where clinicians face similar challenges with malaria diagnostics.

**Trial registration:**

NCT01292707.

## Background

Parasitological confirmation is now recommended for all cases treated as malaria [1]. This reflects recognition of falling malaria prevalence [2] concurrent with overdiagnosis of malaria, with 47% to 95% of patients without malaria parasitaemia being treated with antimalarials in malaria endemic countries [3]. The roll-out of rapid diagnostic tests (RDTs) is hoped to enable this goal in low resource settings through point of care testing [4]. However, availability of RDTs has not meant universal uptake of the tests or adherence to results, with malaria continuing to be overdiagnosed in many settings [3,5]. In Tanzania, overdiagnosis of malaria has been reduced with pilot implementation of RDTs [6-8], but research on routine practice suggests that there is still room for improvement, with almost 50% of febrile patients not being tested and 18% of RDT negative cases being treated with an antimalarial in 2012 [9]. Qualitative studies in Tanzania and other countries suggest that supporting interventions need to address more complex issues than how to do the test [7,10-13], but it is not clear what intervention components are sufficient to support change and be replicable at scale.

The dominant pedagogical approach in Tanzania, including for medical education, has been didactic teaching to large classes with limited engagement of the learner in the process [14]. This is mirrored in clinician-patient interactions, where didactic health ‘education’ is promoted [15]. Student-centred teaching and active, inquiry-based learning have become popular in medical education in Europe and the United States [16], and these constructivist methods have also influenced international education agenda, although their applicability in Tanzania is unclear [17]. The TACT trial at primary healthcare facilities in northeast Tanzania aims to determine whether the addition of small group participatory methods of training for health workers, and communications targeted at patients, are able to improve adherence to national guidelines for use of RDTs and prescription of antimalarial treatment when compared with routine Ministry of Health training.

The processes of development and justification for specific designs of interventions that are trialled in public health are often not published (Chandler *et al.*, under review), hampering development of knowledge of what interventions and theories ‘work’ under different circumstances [18]. In order to develop an intervention that could be tested in a trial and reproducible and sustainable in practice, we undertook a careful process to develop an evidence-based, theoretically informed intervention package to support the uptake of RDTs and adherence to test results. In this paper, we present each stage of our intervention design and a summary of key findings that shaped the design of the intervention. We then present an overview of the programme theory and implementation theory for the intervention components to be tested in this cluster RCT.

## Methods

**Figure 1:**
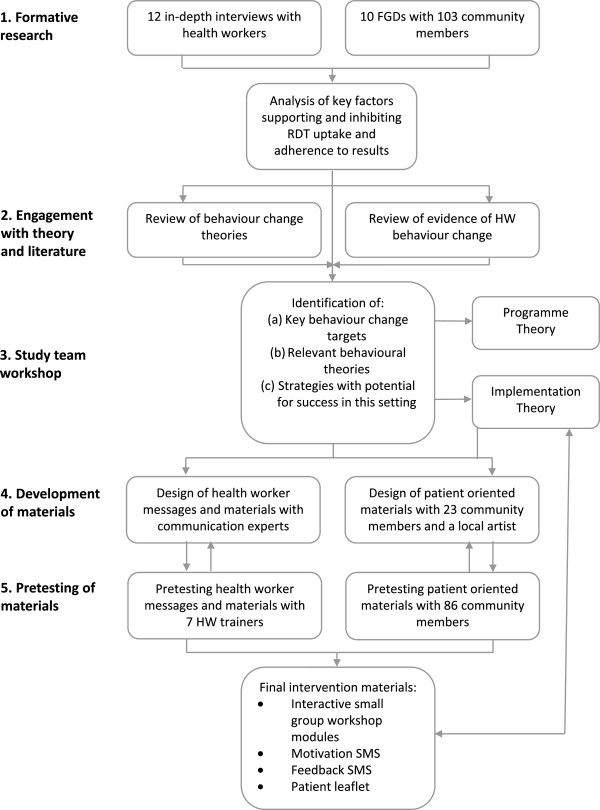
Flow diagram of the process of designing the TACT intervention package.

A summary of the steps involved in the TACT intervention design can be seen in Figure 1. The remit was to develop methods and materials that could be tested in a three-arm intervention trial, comparing (A) routine training of health workers with (B) routine training plus an extended health worker-oriented intervention and (C) routine and extended health worker intervention plus a patient-oriented intervention. The details of the extended health worker- and patient-oriented interventions were not set, although there was interest in applying a broadly constructivist pedagogical approach for arm B. The trial was to be undertaken at dispensaries, which are small primary healthcare centres typically staffed by two or three clinician health workers, including clinical officers and nursing officers, who undertake the work of diagnosis and treatment. The trial was to take place in areas of low and moderate malaria endemicity, where transmission has been declining over the past decade [19].

### Step 1. Formative research

In order to understand the existing scenario of malaria diagnosis and antimalarial use at dispensaries in northeast Tanzania, we undertook formative research with health workers and community members in two districts with different malaria transmission intensities: one in the Kilimanjaro region with low transmission (K-low) and one in the Tanga region with moderate transmission (T-moderate). To address our specific interest in RDTs, we took the opportunity to situate this research at dispensaries where RDTs had been made available as part of a pilot. The pilot’s evaluation showed that availability of RDTs had a limited impact on targeting of antimalarial drugs: many of those receiving antimalarials were not tested (58% in K-low and 30% in T-moderate), and some of those with negative RDT results received antimalarial drugs (23% in K-low and 10% in T-moderate).

Three social scientists in the study team (JM, FN and JK) carried out the formative fieldwork under supervision of CC and after training [20,21]. Fieldwork included informal observations of the set-up and context of dispensaries as well as 12 in-depth interviews with health workers randomly selected from dispensaries with RDTs in the two study areas (six in each area). Following results of previous ethnographic fieldwork with clinicians in the area [10], the topic guide included awareness of a need for change, perception of RDT accuracy, perception of patient preferences, perception of prescribing without testing, influence of peers, and logistics of RDTs. We also carried out 10 focus group discussions (FGDs) with community members living close to dispensaries with RDTs, asking about experiences of illnesses, testing and treatment at different facilities and expectations of malaria treatment. Interviews and FGDs were transcribed and translated from KiSwahili to English. Transcripts were checked for accuracy and then imported into QSR Nvivo (version 8) for coding. Analysis involved thorough reading of each transcript to understand the narratives and meaning conveyed by participants, followed by labelling repeating ideas and organising these into themes. Initial analysis was carried out by JM, FN and JK, with coding cross-checked in NVivo by CC. Higher order themes were developed by CP under supervision of CC.

### Step 2. Review of evidence and engagement with theory

Systematic reviews have been recommended to inform intervention design [22]. We aimed to identify systematic or comprehensive reviews of strategies to improve prescribing behaviour amongst healthcare workers in low income countries in order to guide our choice of strategies for the TACT intervention. We identified existing and ongoing reviews through online databases as well as personal contacts.

It has been argued that theory-based health interventions will be more effective than those with a purely empirical or pragmatic basis [22-24]. A theoretical basis should increase internal coherence of an intervention and when specified explicitly in advance and should enable a better quality of evaluation [25]. It should also enable wider applicability of results [26]. We aimed to engage with theory to formulate the TACT intervention. However, many health behaviour change theories exist, few studies have compared them [27], and evidence appears insufficient for strong recommendations [28]. Furthermore, health behaviour theories often focus on patients and individual cognition [29], and may not apply to health worker practices [16,30]. We therefore aimed to identify theories that had been applied in the past in health worker interventions, particularly in low income countries. We identified reviews of health worker behaviour change that drew on different theories of change, particularly relating to adherence to prescribing guidelines.

### Step 3. Workshop to define intervention approach and content

To decide on the TACT intervention strategy and key content, we followed recommendations of others [31] to hold a structured project workshop to bring together team members involved in data collection and analysis with external collaborators with expert knowledge of the empirical research base and of behaviour change. We set out an intensive one week agenda to (a) review findings from the formative research, empirical literature and behaviour change theory together to identify an approach to guide the intervention design; (b) identify potential strategies for achieving change by listing findings from the formative research, linking each to goals and selecting strategies from literature and theory for each; (c) refine this long list of potential intervention strategies using criteria agreed by the team; and (d) draft outlines of intervention activities and key content.

### Step 4. Design of intervention materials

Once the key methods, learning approach and content of intervention activities were established by the project team, we identified external partners to work with to draft the detail of intervention materials. For the health worker training supplement, we employed communication consultants with expertise in development of training exercises and manuals in East Africa (http://www.wellsense-iphc.com/). For the patient leaflet, we worked with a communications specialist with expertise in development of locally relevant health information (AH) as well as with a local artist. In both cases, drafts were reviewed and revised multiple times through inputs from the research team as well as through a pretesting process.

For the development of the patient leaflet, we took a participatory research approach [32], building on evidence that patient information leaflets are more effective if their presentation is made in a manner that is meaningful to and considerate of potential users [33], and following established methods [34]. Two workshops were held in communities randomly selected from a list surrounding dispensaries where RDTs had been introduced. The first included 10 women with experience of RDTs and the second 10 women with no experience of RDTs. The workshops involved a group discussion about experiences and perceptions of diagnosing malaria, an interactive learning session about RDTs and discussion on the intention of a leaflet to improve uptake and use of RDTs, and a design session to decide on key messages and to draft and revise a story-board for the leaflet. The second workshop built on ideas and pictures from the first. The theoretical approach to the leaflet was to go beyond didactic information-giving common to unsuccessful leaflets [35] to a dialogue format in order to answer, through stories with characters that people could identify with, the questions being asked by people expected to take up RDTs. Principles previously found to be effective in the design of patient information leaflets were followed throughout the process, to ensure cultural relevance [33,36], clarity and number of take-home messages [36], types of pictures used [37], particularly of line drawings for low-literacy readers [38], and attention to complexity of language, organisation and layout [39]. These principles are underpinned by a person-centred approach to information sharing in health [40].

### Step 5. Piloting and pretesting intervention materials

The training materials were piloted alongside a three-day training-of-trainers workshop. Each of the activities drafted in the manuals was piloted with the health workers who would become the trainers and was adapted in this process. The piloting was facilitated by a clinician and a trained actress who together emphasised the principles of reflection, interaction and flexibility in the course of the workshop, in order to enable both learning of future trainers in the methodologies and content of the intervention and also to challenge and adapt the materials. Each of the sections of the modules was tested and adaptations agreed with the future trainers.

We undertook careful pretesting following established methodology [34,41] to ensure leaflets were well understood and relevant to users [42]. Five rounds of interviews were undertaken with a total of 43 pairs of participants in six villages, representing 20 ethnic groups. Pairs were selected to represent a key respondent who had no or limited reading ability, and an ‘assistant’ of their choosing who was able to read well and with whom they had an established relationship, to mimic scenarios where the leaflet might be used in practice. The key respondent was asked to interpret the latest version of the leaflet, with help from their assistant. Each small section of the leaflet was scored using a structured record sheet according to how well the key respondent’s interpretations matched concepts intended to be portrayed, and notes were made on explanations that were required to understand each. Participants were asked for what they understood overall from the leaflet and what could be improved. Scores and participant feedback were reviewed after each round of testing, and changes were made to text, pictures and the flow of the leaflet. Rounds of pretesting ceased when over 90% of the items in the leaflet were understood and respondents could identify the overall messages.

## Results

Here we outline selected key outputs from each stage of our intervention design process.

### Step 1. Outputs of formative research

The qualitative research suggested that the persisting antimalarial overprescription may have been attributable in part to the need to provide a resolution to a disrupted consultation process when RDTs were negative, which was common in the study areas. We describe how RDTs can become a source of conflict with patient expectations and clinicians’ own judgement. We show three routes identified by respondents for resolving these conflicts: employing narratives that undermined the role of RDTs; provision of an alternative diagnosis, preferably with another treatment; or persistence with denial of antimalarial in the hope of shifting expectations over time.

#### RDTs as a source of conflict

Negative RDTs challenged patient expectations from the consultation process. In visiting a health worker, community members expressed an expectation for a process of care that would lead to a cure. This was often expected to involve a prescription for antimalarial drugs, perhaps shaped by the application of the category ‘malaria’ to a wide range of common symptoms. When focus group participants were posed the question*,* ‘how do you know that you have malaria?’, their most common responses were symptoms of joint, waist or back pains and headaches. Interestingly, fever was only mentioned in 4 of 10 focus groups (Table 1). Antimalarials were seen as appropriate treatment for these complaints. Correspondingly, when these symptoms were experienced and malaria treatment denied, respondents described being dissatisfied. In the case of being ‘refused’ antimalarials after a negative RDT, this led to distrust in the science of the test as exemplified in Table 2. These tensions were mirrored by health workers who reported that when results of RDTs conflicted with their or patients’ expectations, they felt pressure to prescribe antimalarials.

**Table 1:** Symptoms of malaria reported by community participants at in the formative research focus group discussions

**Reported symptom of malaria**	**Number of FGDs mentioned in (out of 10)**	**Number of citations in total**
Joint, waist or back pain	8	15
Headache	7	14
Yellow vomit	7	9
Feeling cold	5	6
Fever	4	5
General body pain	3	3
Dizziness	3	3
Body tiredness	2	7

**Table 2:** Key themes emerging from formative research

**Theme**	**Illustrative quotes**
Tension between test results and experience	*‘There is a test at the dispensary [RDT] but it is not trustworthy because you might be sick, or your child is sick, and when you go for a check-up they will tell you that you do not have malaria. So the best thing is to get a trustworthy test.’ (FGD#T3, 30-year-old man, subsistence farmer also a traditional healer)*
	*‘We do not trust the test because everyone who goes for the test is told there is no malaria, but if you are given medication you feel better.’ (FGD#T1, 32-year-old mother of three, female subsistence farmer)*
	*‘A patient came with all malaria signs, but the malaria test was negative. I was in dilemma. I was tempted to give the patient [antimalarial] medication.’ (IDI#T3, male nurse)*
	*‘It affects me very much. Maybe when he or she came from his or her home place he or she had only an expectation to be helped and get the best services. So if you don’t give him or her correct treatment, to be frank it affects me and I extremely lose peace. You have to fulfill what the patient wants. When you don’t fulfill that, even by yourself you will not feel peace in your heart.’ (IDI#T1, female public health nurse)*
	*‘Most of the time they [patients] don’t disturb though a few of them do. If you are not competent in your professional ways they will challenge you and say, “I have come here and I feel malaria completely.” When they test negative, they say “your test is not reliable” … One day a women came and shouted “these tests are fake!” Then I told her make a test somewhere else and come here to compare the results.’ (IDI#T2, female Maternal and Child Health (MCH) Aide)*
Recognition of the need to change practice	*‘If I compare with the present time, I think my prior form of diagnosis was not accurate because we used to see patients who would say they had fever, part of their body was sore, or that their body felt weak, and automatically we would say it was malaria. But since we started using this malaria test, we found that we were giving antimalarial treatment to many more patients than we do now.’ (IDI#K5, female clinical officer in-charge of dispensary)*
	*‘We were not correct, because we were just guessing “that patient has malaria”, and we would give antimalarials. Since we received the test, we just get like three or five children with malaria out of twenty, while before the test we used to say all twenty had malaria because we would give antimalarials to whoever had fever.’ (IDI#T4, female nurse midwife in-charge of dispensary)*
	*‘I am a patient, I go to the doctor, I tell him “give me quinine,” “give me ALU.” I don’t think it is the right way. It is better that the health worker is strong as a professional person who does a test and looks for the disease. Not how we are, so familiar with each other to ask, “give me quinine.”’ (FGD#K6, mother)*
Resolving tensions of tests: questioning the process and rationalising presumptive treatment	*‘When you test and the first patient is negative, the second is negative, then whoever you test results become negative … we met with our colleagues from [a neighbouring dispensary] and they said most of their tests are negative, we tried to find out reasons, for that and we found out that buffer leakage and buffer moving faster than normal were common problems.’ (IDI#T3, male trained nurse)*
	*‘On my side, I will give him or her malaria drugs. I will not feel good [otherwise], I will ask question myself why I should not give this patient malaria drugs? … If I will leave the patient without giving him or her some drug to take it will not be good. I will advise him or her to take malaria drugs … and if he or she will continue being sick, he or she can come back for a check-up again.’ (IDI#K2, female MCH Aide)*
	*‘Sometimes you may find a person who really needs my help with antimalarials, but the test shows no malaria. For that incident I prescribe antimalarials, but very rarely.’ (IDI#T5, female clinical officer in-charge)*
Resolving tensions of tests with alternative diagnoses	*‘If I test a patient as malaria negative, I will not give him antimalarials, but I will give an antibiotic. I have enough experience that sometimes patients come in very sick and I think they have malaria but when you test him you find no malaria, but when you give antibiotic then he recovered.’ (IDI#T6, male assistant clinical officer)*
	*‘I tested myself in the dispensary near our home and thought that it was a joke. She took blood, put it on a testing device. Then after discussion for short moment she told me that I don’t have malaria though she would give me paracetamol tablets as headache pain killers so that I could feel normal. I was also told to avoid a lot of work and get rest. I truly followed the instructions and my condition got better. Maybe it was body tiredness and I felt alright.’ (FGD#K5, 48-year-old male, ward executive officer)*
	*‘I tell them that a fever is not only a symptom of malaria, but other diseases as well. We tell them to take painkillers with them and that they should come back to the dispensary if there is no improvement … Yes in that situation I don’t treat them, but I reassure them that they don’t have malaria.’ (IDI#K5, female clinical officer in-charge)*
Importance of time and experimentation in adopting RDTs	*‘The first problem I had with it was that I could not understand why so many patients were found to be negative. Yet based on what I later found, some patients just had the flu, which is why they had a cough and fever. They never came back with the same symptoms which for me confirmed that this test was accurate.’ (IDI#K5, female clinical officer in-charge)*
	*‘At the beginning, they [patients] do not trust this test, “this person is only playing on the table - no electricity, no any other thing. I don’t think this test is correct!” But as much as we continued to use it, they started to agree with us.’ (IDI#K3, female MCH Aide)*

However, while health workers were keen to meet patient expectations, several distinguished the ‘old’ practice of presumptive treatment of malaria from their new methods with RDTs. These health workers described feeling empowered by the tests, able to provide more accurate diagnosis, and recognising that they had previously overused antimalarial drugs. Community members, too, agreed that the incorporation of tests into clinical practice was a desirable change, but admitted that often they were keen to simply get antimalarial drugs as they had previously expected.

There was therefore a tension between the recognition of a need to change practice and the more immediate fear for health workers that RDTs had the power to undermine their clinical autonomy, becoming a source of conflict with patient expectations.

To deal with these tensions, health workers sometimes employed a narrative that undermined the usefulness, accuracy and relevance of these tests, which conflicts with scientific narratives of accuracy of results from research studies. The experience of some health workers suggested that the use of signs and symptom based diagnosis could be more reliable, and some found their trust in tests undermined by persistent negative results. Rather than taking the test as a single unit in which trust could be placed or not, the tests were deconstructed into different parts and processes, each of which could be questioned for its proper functioning, as exemplified in Table 2. Several health workers questioned whether the RDTs could always ‘see’ the parasites, giving examples of patients who had tested negative but still responded to antimalarial treatment, sometimes after not responding to other treatment first. Some questioned whether RDTs would be accurate if patients had already taken antimalarial drugs. Such narratives rationalised presumptive provision of antimalarial drugs in spite of a negative result, thereby appeasing perceived or actual patient expectations and re-estabishing clinical autonomy.

Respondents also noted that diagnosing the patient with an alternative disease re-established routine dynamics between the clinician and patient. Negative RDT results were more readily accepted once an alternative diagnosis or treatment was provided to the patient. This was echoed by both health workers and community members. Although there was a strong sense that restricting antimalarials to positive RDT cases was considered ‘correct’ practice, only one health worker insisted that he never gave antimalarials to RDT-negative patients. From his perspective, the key to being able to resolve the conflict emerging from the RDT was good communication with patients.

Both health workers and community members’ perceptions of test accuracy was based on the central issue of getting medication and feeling better. Importantly, stories of experiences of ‘experimentation’ when individuals had tried out restricting antimalarials to negative cases appeared to have had an impact on their trust in this practice. This was seen as something that was built over time.

#### Logistical challenges and solutions

Further issues raised by health workers involved the practicalities of doing tests, with health workers reporting that they experienced tests taking longer than the indicated 15 minutes, in some cases closer to 45 minutes. Sometimes multiple tests would produce different results, and respondents wondered whether they were heat sensitive, with results changing with the time of day. Health workers reported challenges with testing small children and patients being able to wait for the right amount of time for test results. Many also complained of the additional workload involved with testing. Informal meetings with colleagues from other dispensaries were reported during health workers’ narratives of acceptance of the tests and integration of processes into their work.

#### Implications of formative results

These results suggested that interventions to support RDT uptake and adherence to results needed to (a) provide support in methods and confidence to communicate results and management to patients; (b) encourage experimentation with restricting antimalarial drugs to RDT positive cases; (c) provide support for technical issues arising in integration of RDTs to the every day realities of their work; and (d) provide opportunities for liaison with colleagues to set and maintain standards. Overall, the results indicated that a multi-level intervention, targeting individual health workers as well as their social interactions with colleagues and patients, as well as the organisational context might be most effective to address the factors shaping existing practice.

### Step 2. Outputs of reviews of evidence and theory

Many reviews have now been undertaken to compare interventions to improve prescriber practices. These have mostly focused on the mode of delivery used to communicate new or existing guidelines, such as printed materials, audit and feedback, reminders, marketing or outreach visits, often in an attempt to improve uptake of research evidence. Findings of such reviews suggest wide variation and mixed effects of each of these methods [43,44] (A. Rowe, personal communication), and authors note the need for better evidence, and for more research to test interventions that tackle organisational, economic and political factors shaping healthcare practices [45]. In reviews of interventions specifically in low resource settings, similar categories of intervention methods have been compared, again with mixed direction and sizes of effect on prescribing practice [46,47], prescription of antimicrobials [48] and for health worker performance in general (A. Rowe, personal communication). One important finding from the ongoing Rowe review is the potential for community oriented interventions to improve provider practices. The overall mixed findings in such reviews provokes the need to analyse the underlying theory of interventions in order to interpret and generalise their findings [44].

We did not identify any reviews that directly compared the effectiveness of interventions based on different theories of health worker behaviour change on prescribing outcomes. Reviews of strategies commonly used to change health worker prescribing behaviour have identified a variety of theories with different foci: individuals, targeting cognition, education and motivation; social interactions, targeting communication, networks, opinion leaders and social learning; organisational context, targeting systems and culture; and wider political and economic contexts, targeting regulation, financing and markets [24,30]. Whilst strategies based on several of these theories have been employed to change health worker prescribing behaviour in low income settings, explicit theory-based interventions have been few, and reviews suggest there is limited evidence upon which to compare effectiveness [30,47]. From a historical perspective, reviews point to an evolution in continuing medical education theory and practice in high-income country settings, which has shifted from acquisition learning to incorporate participatory learning [16], with a greater recognition of the role of experience-based and reflective learning [49] and situational learning, for example through communities of practice [50]. While acknowledging the lack of empirical data, review authors propose that effective intervention design will link theory to empirical research that has identified ‘barriers’ to change [45] and will use a combination of approaches to address multiple levels of influence on practice [24], in particular considering prescribing behaviour in a wider socio-cultural context [47].

### Steps 3 & 4. Outputs of intervention design workshops and activities

#### Programme theory

**Figure 2:**
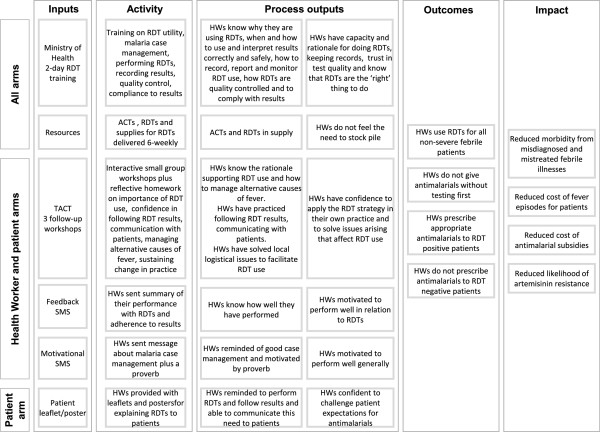
TACT Logic model.

The programme theory for the TACT intervention is depicted in a logic model (Figure 2). The intention for all three packages was to achieve outcomes of RDT use for all febrile patients and adherence to RDT results; a combination of first-line ACT for RDT-positive patients and no antimalarial prescribed to RDT-negative patients. These outcomes were intended to impact morbidity, costs to patients and ACT subsidisers, and a reduction in resistance to artemesinin. The mechanisms by which this would be achieved varied between the three arms of the trial. Each arm was to add further intervention components to the last, in order to evaluate the incremental effects of each package.

##### Control alarm

The basic training intervention, to be implemented in all three arms, was the Ministry of Health’s existing two-day RDT training, intended to equip health workers with knowledge and skills in RDT use and its accompanying practices, with the implicit hypothesis that this would provide them with the capacity and rationale to undertake this work, as well as trust in test quality due to the quality assurance system, and a knowledge that this was the ‘right’ thing to do according to the Ministry of Health.

##### Health worker intervention

An additional package of interventions was designed with the intention to provide further support to health workers in the change to diagnostic-based testing. These were intended to address some of the technical, social, logistical and motivational challenges faced in integrating this new tool and set of guidelines into routine practice. Specifically, these interventions were intended to build health workers’ motivation, skills and confidence to implement the strategy of RDTs in the realities of their own practice. The mode of delivery of the initial intervention in this arm would be a series of interactive learning workshops in small groups. To provide follow-up reinforcement, after several months a series of text messages would be sent to the mobile phones of health workers, firstly in the form of performance feedback, and secondly in the form of motivational messages.

##### Patient intervention

A third intervention was designed with the specific aim to address the perceived expectations of patients for antimalarial drugs. The intention of this intervention was to provide support to health workers in communicating results and treatment decisions to patients who suspect they have malaria. The format for catalysing change was to be a leaflet.

#### Implementation theory

The implementation theory, or TACT intervention change model, is summarised in Table 3.

**Table 3:** TACT intervention model of change

**Stage in the change model**	**Aim of stage**	**Activities in the TACT intervention**
**Stage 1:** Preparing for change	Raising health worker awareness of revised policy in order that individuals and peers consider whether and how to change practice	National MoH Training and TACT Workshop Module 1
**Stage 2:** Enabling change	Providing knowledge, practice and tools that assist health workers to change	TACT Workshops Modules 1, 2 and 3
	Generating perceived patient demand for change	Patient leaflet
**Stage 3:** Reinforcing behaviour	Conducting assessment and feedback on health workers’ changing practice	Self-reflection and group feedback during TACT Workshops
		Text message feedback on practice
		Motivational text messages

##### Control arm

The Ministry of Health two-day training was classroom-based with a short practical at a health facility. Participants were to be provided with a learner manual and a job aid. The training was designed to be standardised by the use of trainer and learner manuals and was intended to take 16 hours in total. Training was to be undertaken by all health workers from each health facility (generally two or three) in groups of 25 to 30 health workers with two Ministry of Health approved trainers, recruited and paid by the study. The content of the training manuals was to be covered by a combination of reading aloud by the facilitator and participants, discussions, question and answer sessions, and by return demonstration. A two-day training of trainers was to be implemented through reading, discussion and return demonstration by the study team.

##### Health worker intervention

The three TACT workshops were to be undertaken every two weeks for a minimum of six hours in total, with the facilitators employed and trained by the research team. The workshops were designed to take place in small groups of three to eight health workers from neighbouring facilities, following the theory that communities of practice with trusted colleagues may support health worker behaviour change [12,50] and that facilitated discussions based on observation and reflection on own practice [51] with small groups of colleagues over time, such as Balint groups, can improve health worker decision-making and relationships with patients [52,53]. The workshop activities and materials were designed to promote a learner-centred and interactive approach, and were based on adult learning steps of reflection on experience, conceptualisation and planning [54]. Each workshop included a range of activities, including physical movement, singing and role plays. The workshops were separated in time in order to allow time for experimentation and reflection between group discussions, and to build trust in colleagues through multiple meetings. After each workshop, participants were asked to carry out reflections on their practice in the form of ‘homework’ that was intended to stimulate short-term change or experimentation, supported by discussion with colleagues during and between workshops, as well as to promote the practice of self-reflection in ongoing practice [55]. The overall approach intended to depart from common teaching practices of hierarchical criticism, where discussion of common problems is often avoided, by building an environment where common goals can be set and challenges discussed openly [56]. The objectives can be found in Table 4. A three-day training-of-trainers workshop was designed to familiarise clinician-trainers, recruited and paid by the study, with interactive methods of learning as well as practicing each of the learning activities in the modules.

**Table 4:** Contents of motivational text messages

**Date/timing**	**Message**	**Proverb**
Monday AM	Prevalence of malaria has declined in Tanzania.	‘The greatest wealth is health’
Monday PM	Malaria is over-diagnosed, use an mRDT for accurate diagnosis.	‘Persistent work triumphs’
Tuesday AM	Not all fever is malaria, look for alternative causes.	‘2 wrongs don’t make a right’
Tuesday PM	Clinical diagnosis of malaria is not reliable.	‘A goal without a plan is just a wish’
Wednesday AM	Perform mRDTs on all patients suspected of having malaria.	‘Actions speak louder than words’
Wednesday PM	mRDTs are reliable. Trust the test.	‘Little by little one walks far’
Thursday AM	All patients with severe illness should be given parenteral treatment and referred immediately to hospital.	‘Better safe than sorry’
Thursday PM	Malaria mRDTs are cost effective if results are followed.	‘A smile you sent, will always return’
Friday AM	Safety precautions are everyone’s responsibility.	‘Prevention is better than cure’
Friday PM	Treat positive tests. Do not treat negative tests.	‘Failing to plan is planning to fail’

The first set of follow-up texts messages were designed to provide a feedback summary to health workers of their health facility’s performance over the past month in terms of RDT use and adherence to results. The second set of text messages were motivational, providing reminders of clinical guidelines together with proverbs (Table 4), which have been used with significant positive impact on performance in Kenya [57].

##### Patient leaflet

Five key areas of content were identified for the patient leaflet after the research workshop and the community participatory workshops: fevers encompass more than malaria; RDTs are available; why tests are trustworthy; experiment in not taking antimalarials after negative test results; health workers should give information to patients. A story was constructed to illustrate these points, depicted in a series of line drawings alongside text and speech bubbles. A one A4 page folded leaflet in black-and-white was considered most feasible for scale-up and interpretation, and the leaflet was designed in English and KiSwahili.

### Step 5. Outputs of piloting and pretesting

The piloting of the training led to a number of adaptations to the activities, implementation style, and notes for the trainers. These included, for example, changes to the way the ‘river walk’ was carried out, to ask participants to guess the dates of malaria guideline changes rather than having these pre-defined, edits to simplify the text of the card game and lamination of the cards, and switching from role-plays in front of the group to paired role-plays in order to create a safer environment for practicing skills.

The five rounds of pretesting of the leaflet incorporated changes including to text, pictures and layout. Clarification of the text included changes such as adding an explanation of the RDT brand name (mRDT), as in ‘In this dispensary, we are now using this new malaria test called malaria Rapid Diagnostic Test in short mRDT’, small changes in text to clarify meaning such as, ‘Joseph, the test is negative’ to ‘Joseph, the test shows there is no malaria,’ and the addition of short lines of text in the body of the leaflet to explain the story. Changes to pictures included adding a health worker doing the test rather than saying she would test the child, removing detail not necessary for the story, such as bushland around the clinic, the characters being easily recognisable by wearing the same clothes in each picture in the series, and the clothing worn being non-specific to any one social group. Arrows linking panels in the story were removed as these caused some confusion, and fewer panels were depicted on each page. Participants were happy with the brand name ‘mRDT’ being used.

The final round of testing of the leaflet found that respondents could interpret the overall messages of the leaflet, and an average of 97.3% of the 120 pictorial and text items of the leaflet were understood (n = 15 pairs), compared with 71.3% of 160 items in the first version (n = 12 pairs).

The health worker trainer and learner manuals and the patient leaflet can be found at http://www.actconsortium.org.

## Discussion

Prescribers in resource poor countries are now being challenged by diagnostic tests that present them with a different philosophy of care. This paper describes an ‘evidence-based’ approach to the design of a health worker intervention and a patient leaflet to support change in practice towards the use of malaria RDTs and adherence to test results. This paper answers the call for clearer descriptions of interventions in order to inform others’ future work [18]. The cluster randomised trial that evaluated these interventions showed that both were more effective than the standard intervention implemented to support RDT introduction, and the trial arm with both interventions was most effective, almost eliminating over treatment of malaria (Cundill *et al.*, forthcoming).

The results of our formative research were central to defining and designing the TACT interventions. These formative findings resonate with reports from elsewhere and as such may suggest that the interventions may be relevant to other settings. For example, RDTs also raised conflicts over their own clinical judgement for clinicians in Ghana [12], as was reported previously with microscopy in Tanzania [10]. The narratives employed by health workers in this study to cope with this conflict, which undermined the usefulness, accuracy and relevance of these tests, have also been reported elsewhere, for example in Uganda [58]. Dealing with this conflict posed by RDTs through identifying alternative disease(s) has also been reported in different settings, while simultaneously drawing attention to the lack of support for diagnosing and treating alternative causes across settings with different levels of malaria transmission [12,58-60]. The experimentation described by both health workers and patients in trying out adherence to test results was also reported in Ghana amongst early adopters of RDTs [12] where the importance of discussion with colleagues in enabling integration of RDTs into routine logistics and practice was also identified. Where such similar experiences and challenges with RDTs are identified, it is possible that a similar or adapted version of the TACT interventions may be effective in increasing uptake of RDTs and adherence to results. Where different challenges are identified, for example concerns about blood taking in malaria RDTs [61], alternative or additional strategies may need to be devised. In any setting, a clear understanding of the current and historical use of malaria diagnostics is likely to be central to supporting the development of effective interventions to support RDT implementation.

This paper demonstrates the time, expertise and resources required for the design of complex interventions, each of which is greater than is implied by the optimistic view that off-the-shelf systematic reviews and behaviour change theories can be directly applied to intervention design [22].

## Conclusions

Our study suggests that evidence-based complex intervention design is possible. We recommend that such evidence includes formative research to understand insider perspectives in context of local priorities and histories as well as empirical research to test materials and methods prior to implementation. Drawing on wider evidence and theory from other interventions will continue to be challenging until the design of interventions is routinely published.

### Ethics

The study was approved by the Ethical Review Boards of the National Institute for Medical Research in Tanzania (NIMRlHQ/R.8cNol. 11/24) and the London School of Hygiene and Tropical Medicine (Approval no. 5877). The trial was registered with clinicaltrials.gov (Identifier #NCT01292707) and was subject to external monitoring from LSHTM to ensure adherence with the protocol.

## Competing interests

The authors declare that they have no competing interests.

## Authors’ contributions

CC, HR and AH conceived the study. JM, FN, CC and JK undertook fieldwork. CC, JM, CP, FN, JK and HM analysed and interpreted the data; CC and CP drafted the manuscript. All authors provided critical revisions and approved the final manuscript.
